# Mouse mandibular–derived osteoclast progenitors have differences in intrinsic properties compared with femoral–derived progenitors

**DOI:** 10.1093/jbmrpl/ziae029

**Published:** 2024-03-04

**Authors:** Rachel Clark, Soo Y Park, Elizabeth W Bradley, Kim Mansky, Amy Tasca

**Affiliations:** Department of Developmental and Surgical Sciences, Oral Biology Graduate Program, University of Minnesota School of Dentistry, Minneapolis, MN 55455, United States; School of Dentistry, University of Minnesota, Minneapolis, MN 55455, United States; Department of Orthopedic Surgery, University of Minnesota, Minneapolis, MN 55455, United States; Division of Orthodontics, Department of Developmental and Surgical Sciences, University of Minnesota School of Dentistry Minneapolis, Minneapolis, MN 55455, United States; Division of Orthodontics, Department of Developmental and Surgical Sciences, University of Minnesota School of Dentistry Minneapolis, Minneapolis, MN 55455, United States

**Keywords:** osteoclasts, transcription factors, mandible, precursors, proliferation

## Abstract

Craniofacial osteoclasts are essential for site–specific processes such as alveolar bone resorption, tooth eruption, and orthodontic tooth movement. Much of the current understanding of osteoclast development and function comes from studies using long bone–derived cells. Minimal investigation has been done to explore skeletal site differences. The overall goal of this study was to determine if mandibular– and femoral–derived osteoclasts represent distinct populations. To test this hypothesis, bone marrow cells were initially analyzed from the mandible and femur of 2–month–old mice. It was shown that mandibular–derived osteoclasts have enhanced size (mm^2^) compared with femoral–derived osteoclasts. Since bone marrow macrophages are a heterogenous population, we additionally selected for monocytes and demonstrated that mandibular–derived monocytes also form osteoclasts with increased size compared with femoral–derived monocytes. Osteoclast precursor populations from both skeletal sites were analyzed by flow cytometry. A newly described Ly6C^High+^ population as well as the Ly6C^int^ population was increased in the mandibular–derived cells. The difference in differentiation potential between monocyte cultures suggests that the increase in the Ly6C^High+^ population may explain the enhanced differentiation potential in mandibular–derived cells. Monocyte genes such as *Pu.1*, *C/ebp-a,* and *Prdm1* are increased in expression in mandibular–derived monocytes compared with femoral–derived monocytes. As expected with enhanced differentiation, osteoclast genes including *Nfatc1, Dc-stamp, Ctsk*, and *Rank* are upregulated in mandibular–derived osteoclast precursors. Future studies will determine how changes in the environment of the mandible lead to changes in percentages of osteoclast progenitors and their differentiation potential.

## Graphical Abstract

**Graphical Abstract:**
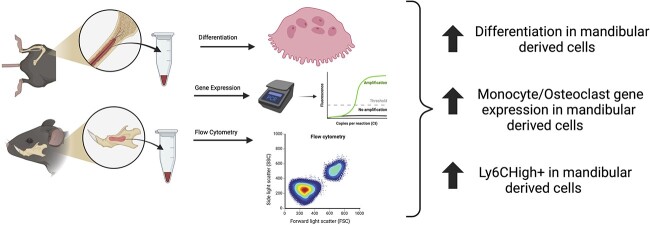

## Introduction

Osteoclasts are large, multinucleated cells of hematopoietic origin responsible for bone resorption.^1^ They arise from fusion of osteoclast precursor cells from the myeloid/monocyte population expressing both CD11b/Ly6C cell surface proteins.^2^^,^^3^ Bone resorption by osteoclasts crucially maintains skeletal homeostasis and coordinates biological processes such as growth and bone repair. Osteoclast activity within the craniofacial region orchestrates critical processes such as tooth eruption, skull modeling, orthodontic tooth movement, as well as craniofacial bone healing and regeneration during periodontal disease and osseointegration of dental implants.^4–6^ Historically, homogeny of the osteoclast populations throughout the body was assumed because of the limited capacity for isolation and limited exploration of these populations at the level of specific skeletal sites.^7^ In contrast, the advent of single–cell sequencing techniques and advanced cell isolation and culturing has unveiled considerable heterogeneity among cell types across the body. Importantly, this includes heterogeneity between osteoclast populations at varying skeletal sites, which could significantly advance our understanding of bone biology.^8^

Previous in vitro studies suggest the size, resorptive abilities, and gene expression of osteoclasts derived from marrow within the mandible differ from that of osteoclasts derived from femoral marrow cells.^9–13^ Although there is agreement that osteoclasts derived from the bone marrow cells within the calvaria and the mandible exhibit different characteristics than those derived from bone marrow cells of the appendicular skeleton, variability exists between the conclusions drawn about differences among the multiple studies. Additionally, exploration of gene expression variances that contribute to these differences and mechanistic suggestions for the source of this variation are few. Furthermore, the referenced studies examine osteoclasts populations at the largely differentiated stage, with little consideration given for differences observed at earlier time points during osteoclastogenesis.

In our current study, we explored the variation in osteoclastogenic potential between mandibular– and femoral–derived bone marrow cells at early time points of osteoclast differentiation. Since bone marrow macrophages (BMMs) are a heterogenous population, we also selected for monocytes to explore their contribution to osteoclastogenic differences observed at these early time points. The aim was to uncover potential mechanisms for observed differences by identifying precursor populations by flow cytometry and analyzing osteoclast gene expression by quantitative PCR (RT-qPCR). The results of the study will further our understanding of early developmental differences in osteoclast populations of the mandibular and femoral skeletal sites, as well as begin to elucidate possible mechanistic drivers of these discernable differences.

## Materials and methods

### Ethics

The use and care of mice used in this study were reviewed and approved by the University of Minnesota Institutional Animal Care and Use Committee, IACUC protocol number 2104-39006A. Euthanasia was performed by CO_2_ inhalation. This study complies with the updated ARRIVE guidelines.

### Harvest and culture of primary osteoclasts from femoral and mandibular bone marrow

Primary BMMs were harvested from the femurs and tibiae of 2–month–old male and female C57Bl/6 J mice. Femurs and tibiae were dissected, and adherent tissue was removed. The ends of the bones were cut, and the marrow was flushed from the inner compartments. Red blood cells were lysed from the flushed bone marrow tissue with RBC lysis buffer (150 mM NH4Cl, 10 mM KHCO3, 0.1 mM EDTA, pH 7.4) and the remaining cells were plated and cultured overnight in 100 mm tissue culture dishes (TPP, MidSci) in osteoclast media (phenol red-free alpha-MEM (Gibco, catalog # 41061-037) with 5% fetal bovine serum (Atlanta Biologicals, catalog # S12450), 25 units/mL penicillin/streptomycin (Invitrogen, catalog # 15140-122), 400 mM L-Glutamine (Invitrogen, catalog # SH30034.01), and supplemented with 1.5% CMG 14-12 supernatant (culture supernatant containing macrophage–colony stimulating factor (M-CSF), Dr Sunao Takeshita, Nagoya City University, Nagoya, Japan)). Mandibles were dissected in two portions, a right and left side, and adherent tissue was removed. The molar teeth of each mandibular section were extracted, and tooth sockets were flushed. The condylar and incisal portion of the mandible was cut, and the marrow space was flushed from the inner compartments. Similar to the femoral cells, bone marrow cells were cultured overnight in osteoclast media supplemented with 1.5% CMG 14-12 supernatant. Twenty-four hours later the non–adherent cell population, including osteoclast precursor cells, was separated from the adherent cells and replated in 24–well plates (TPP, MidSci) at 2 × 10^4^ cells/cm^2^ in osteoclast media supplemented with 1.5% CMG 14-12 culture supernatant. Two days later, cells were refed with 1.5% CMG 14-12 culture supernatant and 5 ng/mL of receptor activator of NF-kB ligand (RANKL) (R and D Systems, catalog #462-TEC-010) to stimulate osteoclast differentiation. Cultures were fed every other day for up to 4 days for differentiation experiments.

### Harvest and culture of primary osteoclasts from monocytes of the femoral or mandibular bone marrow

Primary BMMs were harvested from the femurs and mandible of 2–month–old male and female C57Bl/6 J mice as described in the femur and mandible bone marrow section. Bone marrow monocyte selection was performed on total bone marrow. Monocytes were selected following the protocol of the mouse monocyte isolation kit (Miltenyibiotec, catalog #130-100-629). Briefly, flushed cells were pelleted and resuspended in MACS buffer (phosphate–buffered saline (PBS), pH 7.2, 0.5% bovine serum albumin, 2 mM EDTA). The cells then were incubated with FCR Blocking Reagent and Monocyte Biotin–Antibody Cocktail. After 5 min the cells were rinsed with MACS buffer, pelleted, and resuspended in MACS buffer. The cells were incubated with Anti–Biotin Microbeads for 10 min. After this incubation period the cells were applied to the MACS columns in a magnetic field. Flow through was collected as isolated monocytes. The columns were rinsed three times to collect unbound monocyte cells. The monocyte cells were plated in 24–well plates (TPP, MidSci) at 2 × 10^4^ cells/cm^2^ in osteoclast media supplemented with 1.5% CMG 14-12 culture supernatant overnight. The next day cells were refed with 1.5% CMG 14-12 culture supernatant and 5 ng/mL RANKL (R and D Systems, catalog #462-TEC-010) to stimulate osteoclast differentiation. Cultures were fed every other day for up to 4 days for differentiation experiments.

### Tartrate resistant acid phosphatase staining of osteoclast cultures

Primary osteoclasts were fixed with 4% paraformaldehyde and washed with PBS. The cells were then stained for tartrate resistant acid phosphatase (TRAP) expression using Naphthol AS-MX phosphate and Fast Violet LB salt according to protocol described in BD Biosciences Technical Bulletin #445. Cells were then imaged and photographed with light microscopy at 4× magnification and measurements were analyzed from images (*N* ≥ 3) using NIH ImageJ software.

### C‌CK-8 assay

Femoral and mandibular selected monocytes were plated in 96–well plates in 100 μL of osteoclast media and 1.5% CMG14-12 conditioned media and incubated for indicated time. Ten microliter of CCK-8 solution (Sigma-Aldrich, catalog #96992) was added to each well. Cells were incubated at 37 °C for 3 h. Absorbance was measured at 450 nm.

### Real–time qRT-PCR

RNA was harvested from both selected monocytes and primary osteoclast cells plated in triplicate using Trizol Reagent (Ambion, Life Technologies, catalog # 15596018) and quantified using UV spectroscopy. cDNA was then prepared from 1 μg RNA using the iScript cDNA Synthesis Kit (Bio-Rad, catalog #1708891) as per the manufacturer’s protocol. Quantitative real–time PCR was performed in duplicate using the MyiQ Single Color Real–Time PCR Detection System (Bio-Rad, catalog #172-5121). Each 20 μl reaction contained 1 μl cDNA, 10 μl iTaq Universal Sybr Green Supermix, and 500 nM forward and reverse primers. The PCR conditions were as follows: 95 °C for 3 min, and the 40 cycles of 94 °C for 15 s, 56 °C for 30 s, and 72 °C for 30 s, followed by melting curve analysis (95 °C for 5 s, 65 °C for 5 s, and then 65-95 °C with 0.5 °C increase every 5 s). Primer sequences are listed in Table 1.

**Table 1:** Primers.

**Primer name**	**5’-3’ sequence**
*Cebp-α* Forward	TGG ACA AGA ACA GCA ACG AGT AC
*Cebp-α* Reverse	GCA GTT GCC CAT GGC CTT GAC
*c-Fos* Forward	CCA AGC GGA GAC AGA TCA ACT T
*c-Fos* Reverse	TCC AGT TTT TCC TTC TCT TTC AGC AGA
*c-Jun* Forward	TCC CCT ATC GAC ATG GAG TC
*c-Jun* Reverse	TGA GTT GGC ACC CAC TGT TA
*Csfr1* Forward	TGC TAA AGT CCA CGG CTC AT
*Csfr1* Reverse	TCG GAG AAA GTT GAG ATG GTG T
*Ctsk* Forward	AGG GAA GCA AGC ACT GGA TA
*Ctsk* Reverse	GCT GGC TGG AAT CAC ATC TT
*Dc-stamp* Forward	CAG ACT CCC AAA TGC TGG AT
*Dc-stamp* Reverse	CTT GTG GAG GAA CCT AAG CG
*Hprt* Forward	GAG GAG TCC TGT TGA TGT TGC CAG
*Hprt* Reverse	GGC TGG CCT ATA GGC TCA TAG TGC
*Nfatc1* Forward	TCA TCC TGT CCA ACA CCA AA
*Nfatc1* Reverse	TCA CCC TGG TGT TCT TCC TC
*Prdm1* Forward	AGT TCC CAA GAA TGC CAA C
*Prdm1* Reverse	TTT CTC CTC ATT AAA GCC ATC AA
*Pu.1* Forward	GGC AGC AAG AAA AAG ATT CG
*Pu.1* Reverse	TTT CTT CAC CTC GCC TGT CT
*Rank* Forward	CCA GGA CAG GGC TGA TGA GAA
*Rank* Reverse	TGG CTG ACA TAC ACC ACG ATG A

### Caspase assay

Primary BMMs were analyzed using Promega Caspase-Glo 3/7 Assay (Promega, Catalog #G8090) according to protocol outlined by Promega technical bulletin #TB323 and 24 h after plating to measure apoptosis of the in vitro cultures as per the manufacturer's protocol with minor changes. Cells were seeded at 100 000 cells per well in 24–well plates in quadruplicate. On the indicated day, room temperature Caspase-Glo 3/7 reagent was added to each well to lyse cells for 30 min at room temperature. After lysis, caspase activity for each sample was measured using the GloMax20/20 Luminometer (Promega). Since Caspase-Glo 3/7 activity is highly dependent on the number of cells in each reaction (see manufacturer's datasheet), the average nuclei number per culture was assessed in additional cells plated and cultured in parallel.

### Flow cytometry of osteoclast precursors

Bone marrow cells were isolated from femora, tibiae, and mandibles as described above. Red blood cells were lysed using ACK lysis buffer (Thermo Fisher) and subsequently washed with cold 1X PBS. CD11b + cells were positively selected for using CD11b microbeads and magnetic column (Miltenyi Biotec, catalog #130-097-142) following manufacturer’s instructions. Isolated CD11b + cells were stained with Zombie Aqua viability dye (Biolegend, catalog # 423101) for 10 min at room temperature in the dark. Cells were then incubated with Mouse BD FC block (CD16/32, Clone 93, BD Biosciences, Catalog # 553142) for 15 min at 4 °C in the dark. Anti–Ly-6C Brilliant Violet 785 (Biolegend, catalog #128041) antibody was added to cells and allowed to incubate at 4 °C in the dark for 30 min. Cells were washed twice with ice–cold PBS and transferred to flow cytometry tubes. Flow cytometry was performed using the four laser BD LSR II Flow Cytometer (BD Biosciences) and the BD FacsDiva Software (BDBiosciences). Single color–stained beads (BD Biosciences, catalog # BDB552845) were used for compensation controls. Flow data were analyzed using FlowJo software (FlowJo, LLC).

### Statistical analysis

All experiments were completed in triplicate and performed with at least three biological replicates. The data shown are representative of the mean ± SD of all experiments. Student’s *t*-test or a one–way ANOVA analysis followed by a Tukey’s multiple comparison test were used to compare data using GraphPad Prism.

## Results

### Mandibular–derived BMMs have increased potential for osteoclast differentiation.

**Figure 1:**
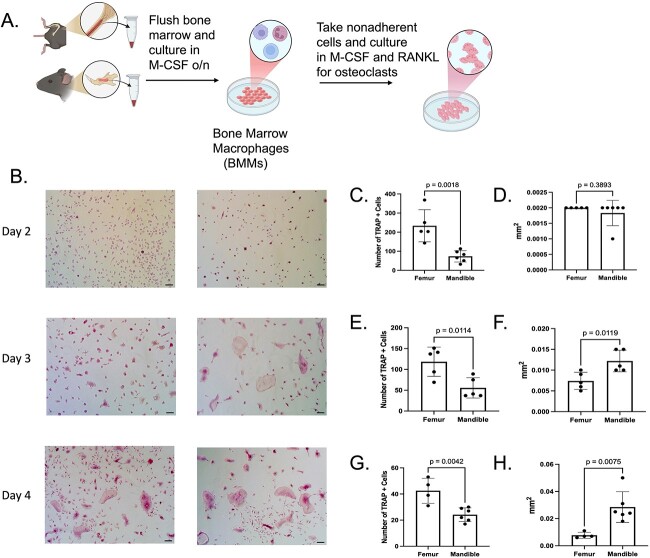
Bone marrow–derived macrophages (BMMs) from the mandible show increased osteoclast differentiation potential. BMMs were flushed from mandibular or femoral bone marrow of 2–month–old C57B1/6 J mice. (A) Schematic illustrating the isolation procedure of BMMs for osteoclast cultures; (B) representative TRAP–stained images of BMMs differentiated with M-CSF and RANK-L for 2-4 days. Quantification of TRAP–stained images measuring number of TRAP positive osteoclasts at (C) 2, (E) 3, and (G) 4 days (*N* ≥ 3). Quantification of TRAP–stained images measuring size of TRAP positive osteoclasts at (D) 2, (F) 3, and (H) 4 days (*N* ≥ 3). Samples were compared using Student’s *t*-test. Scale bar is 10 mm.

To compare osteoclast differentiation between mandibular– and femoral–derived BMMs, bone marrow from each skeletal site was flushed and cells were plated for culture. To stimulate osteoclast differentiation, cells were treated with M-CSF and RANK (Figure 1A). Both size (mm^2^) and number of TRAP positive osteoclasts were measured on days 2-4 (D2-D4) after RANKL stimulation. On day 2, mandibular–derived osteoclasts were reduced in number compared with femoral–derived cells with an average of 73 cells in the mandible as compared with an average of 233 cells in the femur, with no significant difference in size (mm^2^) (Figure 1B-D). Higher magnification of day 2 femoral and mandibular cells demonstrates the presence of binuclear cells in the mandibular cultures (Figure S1). On days 3 and 4 mandibular–derived osteoclasts exhibited increased size as compared with the femoral–derived osteoclasts with an average cell size of 0.0122 (day 3) and 0.0285 mm^2^ (day 4) in the mandible as compared with 0.0074 (day 3) and 0.0077 mm^2^ (day 4) in the femur (Figure 1F and H). While TRAP+ multinuclear cells derived from the femur were smaller at days 3 and 4, they were more numerous compared with mandible–derived cells (Figure 1E and G).

### Mandibular–derived monocytes form larger osteoclasts compared with femoral–derived monocytes

**Figure 2:**
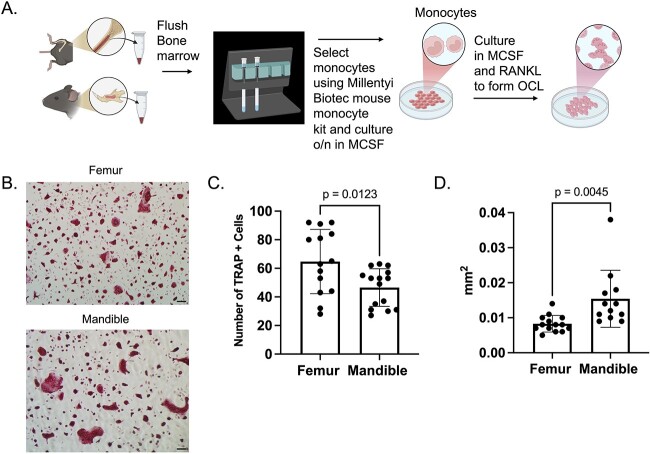
Monocytes isolated from mandible bone marrow have enhanced osteoclast differentiation. Bone marrow cells were flushed from mandibular and femoral bone and monocytes were selected for osteoclast cultures from 2–month–old mice. (A) Schematic illustrating procedure for selecting monocytes from bone marrow for osteoclast cultures; (B) representative TRAP–stained images of monocytes differentiated into osteoclasts in the presence of M-CSF and RANKL for 4 days. (C) Quantification of the number of TRAP positive multinuclear cells (*N* ≥ 3). (D) Quantification of size of TRAP positive multinuclear cells. Scale bar is 10 mm.

As non–adherent bone marrow cultures grown in M-CSF overnight are heterogenous in nature, we selected monocytes from mandibular– and femoral–derived bone marrow to culture into osteoclasts. Bone marrow from each skeletal site was flushed, monocytes were negatively selected and plated for osteoclast differentiation (Figure 2A). To verify isolation of monocytes, cells were analyzed for Ly6C expression by flow cytometry. We were able to demonstrate that we had a population of 80.6% of Ly6C^+^ cells after monocyte selection (Figure S2A-B). To stimulate osteoclast differentiation, cells were treated with M-CSF and RANKL. Both size (mm^2^) and number of TRAP positive osteoclasts were measured on day 3 (Figure 2B-D). On day 3, mandibular–derived osteoclasts were reduced in number compared with femoral–derived cells with an average of 46 cells in the mandible as compared with an average of 64 cells in the femoral–derived cells (Figure 2C). However, as seen with BMMs, mandible selected monocytes exhibited increased size as compared with the femoral–derived osteoclasts with an average cell size of 0.01542 mm^2^ in the mandible as compared with 0.008286 mm^2^ in the femoral–derived cells (Figure 2D). These data suggest that differentiation potential is skeletal site specific.

### Mandibular–derived CD11b + cells exhibit an increase in Ly6CHigh + populations

**Figure 3:**
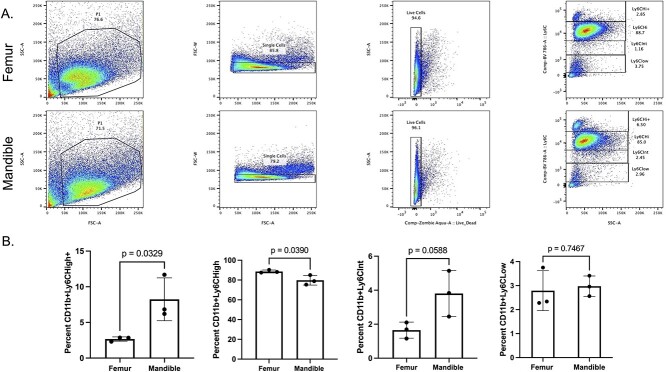
Mandibular–derived CD11b + cells exhibit an increase in Ly6C^High+^ and Ly6C^Int^ populations. CD11b + cells were positively selected for using CD11b microbeads and magnetic column. Isolated CD11b^+^ cells were stained with Zombie Aqua viability dye and anti–Ly-6C Brilliant Violet 785. Flow cytometry was performed using the four laser BD LSR II Flow Cytometer and the BD FacsDiva Software. Single color–stained beads were used for compensation controls. Flow data were analyzed using FlowJo software. (A) Representative image of analysis. (B) Graphed quantification of population distributions from three biologically independent samples. Samples were compared using Student’s *t*-test.

To determine if precursor cell population distributions impact the differences, we observed in osteoclastic differentiation potential both in BMMs and monocyte cultures (Figures 1-2), we examined precursor populations of the mandibular– and femoral–derived bone marrow by flow cytometry. To narrow our search into cells that have osteoclastogenic potential, the cells were first separated using CD11b microbeads for positive selection. CD11b^+^ cells include neutrophils, monocytes, macrophages and certain dendritic cell populations.^14^ Positively selected cells were then stained with an anti–Ly6C antibody. Previously reports established that Ly6C^High^ population has the highest osteoclastogenic potential.^3^ In our data, we observed an additional Ly6C^High^ population not previously reported. We have termed these cells Ly6C^High+^. Our data showed that bone marrow cells from the mandible exhibited a higher percentage of Ly6C^High+^ cells, with 6.50% in the mandible as compared with 2.85% in the femur (Figure 3B, Ly6C^High+^). Femur–derived CD11b^+^ cells contained significantly more Ly6C^High^ cells compared with mandible–derived cells (Figure 3B, Ly6C^High^). Our reported percentage of the Ly6C^High^ population agrees with previously reported percentages of this population in the bone marrow.^3^ Surprisingly, mandibular–derived cells demonstrated a significantly higher percentage of Ly6C^Int^ cells with 2.45% in the mandible as compared with 1.16% in the femur (Figure 3B, Ly6C^int^). Previous studies demonstrated that this intermediate group has the potential to differentiate into osteoclasts in the absence of *Irf8* expression*,* an osteoclast inhibitor.^3^ Future studies will strive to understand the epigenetic and transcriptional changes in the Ly6C^High+^ and Ly6C^int^ populations between the femur and mandible skeletal sites.

### Mandibular–derived osteoclasts proliferate less than femoral–derived osteoclasts and demonstrate no increase in apoptotic activity

**Figure 4:**
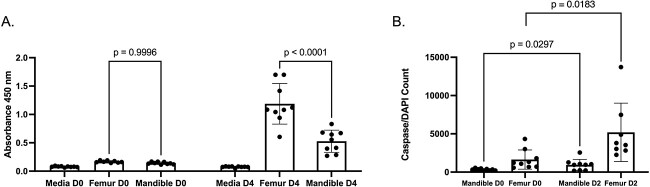
Mandibular–derived osteoclast precursors proliferate less than femoral–derived osteoclast precursors and do not exhibit an increase in apoptosis activity. Bone marrow cells were flushed from the mandible and femur of 2–month–old C57Bl/6 mice and monocytes were selected. (A) Monocytes were grown in M-CSF for 4 days. CCK-8 activity was measured at 450 nm at plating (day 0) or after 4 days in M-CSF. (B) Osteoclast precursors were grown in M-CSF (day 0) or M-CSF and RANKL for 2 days (day 2). Apoptosis activity was quantified at day 0 or 2 using Promega Caspase-Glo 3/7 Assay. Graphed data represent Caspase-Glo3/7 activity normalized to the average number of nuclei in each culture condition (*N* ≥ 3). Samples were compared using Student’s *t*-test.

To further characterize differences between the mandible– and femur–derived osteoclast populations, we decided to determine if there were changes in proliferation of osteoclast precursors. Changes in the proliferation of osteoclast precursors may explain differences in osteoclast differentiation we observe between BMMs isolated from opposing skeletal sites as it has been shown that osteoclast precursors exit the cell cycle before beginning the fusion process.^15^^,^^16^ CCK-8 activity between femoral– and mandibular–derived cells was measured in monocytes and BMMs (Figures 4A and S3A). We observed no significant difference in CCK-8 activity at day 0 between femur– and mandible–derived cells. However, in both monocytes and BMMs, a significant difference in CCK-8 activity was observed at day 4 with the femoral–derived cells having a 40% higher CCK-8 activity compared with mandibular–derived cells (Figures 4A and S3). To ensure that diminished proliferation was not because of an increase in apoptotic activity, we measured the apoptotic activity of the BMMs from each site at days 0 and 2. We observed a significant increase in apoptotic activity in both the mandibular–derived BMMs and the femoral–derived BMMs after 2 days in M-CSF and RANKL (Figure 4B). These data suggest that increased osteoclast differentiation potential of mandible–derived cells does not arise from increased proliferation or decreased apoptosis of osteoclast precursors.

### Mandibular–derived osteoclast progenitors exhibit an increase in gene expression in osteoclast and macrophage marker genes

**Figure 5:**
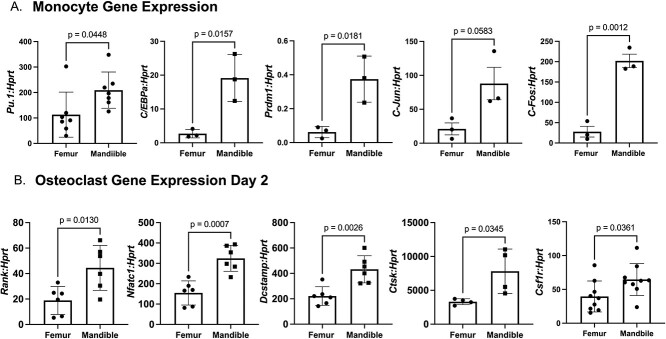
Monocytes and osteoclasts derived from mandibular bone marrow exhibit increased gene expression. qRT-PCR comparing expression of (A) monocyte genes *PU.1*, *C/epb-a*, *Prdm1*, *C-Jun*, and *C-Fos* and (B) osteoclast genes *Rank*, *Nfatc1*, *Dcstamp*, *Ctsk*, and *Csf1r*. Data shown are from at least three independent experiments in which gene expression was measured. Each PCR reaction was performed in duplicate. Graphed data are relative to expression of *Hprt*. Samples were compared using Student’s *t*-test.

Increased osteoclast differentiation observed in the mandibular–derived monocytes suggests that there might be differences in gene expression between the two skeletal sites. We determined gene expression in monocytes from the mandible and the femur. Monocytes were isolated from the bone marrow as described for differentiation experiments (Figure 2). We saw a significant increase in transcription factors *Pu.1*, *Cebp-α*, *c-Jun*, and *c-Fos* in mandible–derived monocytes compared with femur–derived monocyte (Figure 5A). All the transcription factors tested have been shown to promote osteoclast differentiation.^17–20^ Blimp1, encoded by *Prdm1*, functions as a transcriptional repressor of *Irf8* and *MafB*, inhibitors of osteoclast differentiation. Overexpression of Blimp1 leads to increased osteoclast differentiation.^21^ Our data suggest mandible–derived osteoclast progenitors have increased expression of early stage osteoclast differentiation transcription factors which may explain the enhanced osteoclast differentiation seen in the mandible–derived monocytes. We also measured gene expression differences of genes characteristic for osteoclast differentiation and function. In terms of osteoclast genes, we observed significantly increased expression of *Rank*, *Nfatc1*, *Dcstamp*, *Ctsk*, and *Csf1r* in the mandibular–derived BMMs at day 2 compared with femoral–derived cells (Figure 5B). Collectively, these data support our observation of enhanced osteoclast differentiation of mandibular–derived cells (Figures 1-2).

## Discussion

The skeletal site location, perhaps through environmental factors, influences the function of cells that exist at that site. In the femur, osteoclasts orchestrate bone modeling and remodeling while in the mandible besides modeling and remodeling specialized processes unique to the mandible and maxilla such as tooth eruption also require osteoclasts. Defining the differences between these cell populations has implications for our understanding of craniofacial growth and development, as well as our grasp of wound healing and craniofacial site–specific conditions such as periodontal disease and osteonecrosis of the jaw.

Although a plethora of studies describe the regulation of osteoclasts in long bones, this does not extend to mandibular–derived osteoclasts. While several studies have taken on the task of trying to address this gap in knowledge, conclusions drawn by these studies are variable and few explore mechanistic processes.^9–13^ In this study, we show that mandibular–derived osteoclasts cultured from either BMMs or monocytes are larger than their femoral–derived counterparts and proliferate less compared with femur–derived osteoclasts. We demonstrate that our monocyte cultures contained primarily Ly6C^+^ cells (Figure S2). Osteoclasts primarily derive from the Ly6C^+^ monocyte lineage^3^; however, bulk RNA-Seq and ChIP-SEQ studies have demonstrated that Ly6C^High^ and Ly6C^Int^ populations are transcriptionally and epigenetically programmed to differentiate into osteoclasts compared with Ly6C^low^ population.^3^ We show that mandible–derived CD11b^+^ cells have an increase in the percentage of Ly6C^Int^ cells and a decrease in the percentage in Ly6C^High^ cells compared with femur–derived CD11b + cells. This trend is similar to the changes in percentages of Ly6C cells seen in *Irf8* conditional knockout mice in which the authors demonstrate that increased osteoclast differentiation observed in those mice was because of the an increase in the percentage Ly6C^Int^ population in the cKO compared with WT mice as well as increase in osteoclast differentiation occurring from cells in the Ly6C^int^ population.^3^ Our cell culture and flow cytometry data suggest that it not only Ly6C^High^ cells but also possibly the Ly6C^int^ cells that are responsible for the differences that we determined between mandible– and femur–derived osteoclasts. However, a limitation to our study is that we did not do any unbiased analysis of the transcriptome of the different Ly6C populations between the two skeletal sites. Future studies will entail transcriptome and epigenetic analysis of the Ly6C^High,^ Ly6C^int^ as well as our newly identified Ly6C^High+^ populations from mandible–derived bone marrow to determine if these populations have altered expression of osteoclast genes which may account for increased osteoclast differentiation determined from mandible–derived bone marrow.

Several studies have suggested that RANKL induced osteoclast differentiation regulates cell proliferation through cyclin dependent protein kinases and cyclin dependent kinase inhibitors.^15^^,^^16^ Our proliferation data that mandible–derived osteoclast precursors proliferate less may suggest that mandible–derived osteoclast precursors have already begun the molecular process of differentiation compared with femur–derived precursors. An interesting question is why mandible–derived osteoclast precursors are “primed” for differentiation compared with femur precursors? Additionally, mandibular–derived osteoclasts demonstrate increased gene expression of key osteoclast marker genes. We also show that mandibular–derived bone marrow cells exhibit increased percentages of CD11b^+^/Ly6C^High+^ and CD11b^+^/Ly6C^Int^ populations. Taken together, our results strongly support our hypothesis that mandibular–derived osteoclast populations possess increased osteoclastogenic potential compared with femoral–derived osteoclast precursors and that this increased potential may be because of changes in transcriptional and epigenetic status of mandible–derived osteoclast precursors. It has yet to be determined if the changes in gene expression are because of shifts in osteoclast precursor populations. One caveat to our studies is that we were unable to measure bone resorption with our BMMs or monocyte cultures, so we are not able to determine if the enhancements in differentiation correlate with changes in osteoclast activity.

In our study, we investigated the difference between these cells in young 2–month–old mice, but further exploration into aged mice should be considered. In human and animal studies, osteoclast activity is enhanced both as an individual ages and because of altered hormonal status^22–26^; however, little is known about if osteoclast activity changes in the mandible. Previous results from in vivo human and animal studies of craniofacial bone are not clear if bone is lost with hormonal status and age similar to that seen with long bones.^27^^,^^28^ Now that we have performed an analysis of the osteoclast precursors in the mandible, an evaluation of the impact of age on these populations will further inform our understanding of the age–related changes within different skeletal sites via osteoclast differentiation.

Future studies will aim to determine the molecular signature of CD11b^+^ populations including osteoclast precursors using single–cell RNA sequencing of this heterogenous population of cells. Previously conducted single–cell analysis of the whole bone marrow of mandible– and femur–derived cells suggests that the mandible has a more active immune environment and a higher proportion of mature immune cells.^29^ This study went on to demonstrate that mandible–derived monocyte/macrophages express higher levels of the osteogenic factor *oncostatin M* (*Osm*) compared with femur–derived monocyte/macrophage.^29^ Additionally, a study analyzing differences in myeloid cell heterogeneity between mandibular and femoral bone identified differences in transcriptome profiles in myeloid populations found in the two skeletal sites.^30^ The authors suggest that these differences may account for differences in osteoclast differentiation observed between mandible– and femur–derived bone marrow under physiological conditions.^30^ Our results, along with these single–cell sequencing studies, suggest that the mandibular–derived osteoclasts are a distinct population of osteoclasts compared with femoral–derived cells.

## Supplementary Material

Supplemental_Figure_1_for_JBMR_Plus_ziae029

Supplemental_figure_2_for_JBMR_Plus_ziae029

Supplemental_figure_3_for_JBMR_Plus_ziae029

## Data Availability

Data are available upon request.
