# Supplementary figures and images for: Mouse mandibular–derived osteoclast progenitors have differences in intrinsic properties compared with femoral–derived progenitors
Source: JBMR Plus. 2024 Mar 4;8(5):ziae029. doi: 10.1093/jbmrpl/ziae029 (PMC11008737; doi:10.1093/jbmrpl/ziae029)

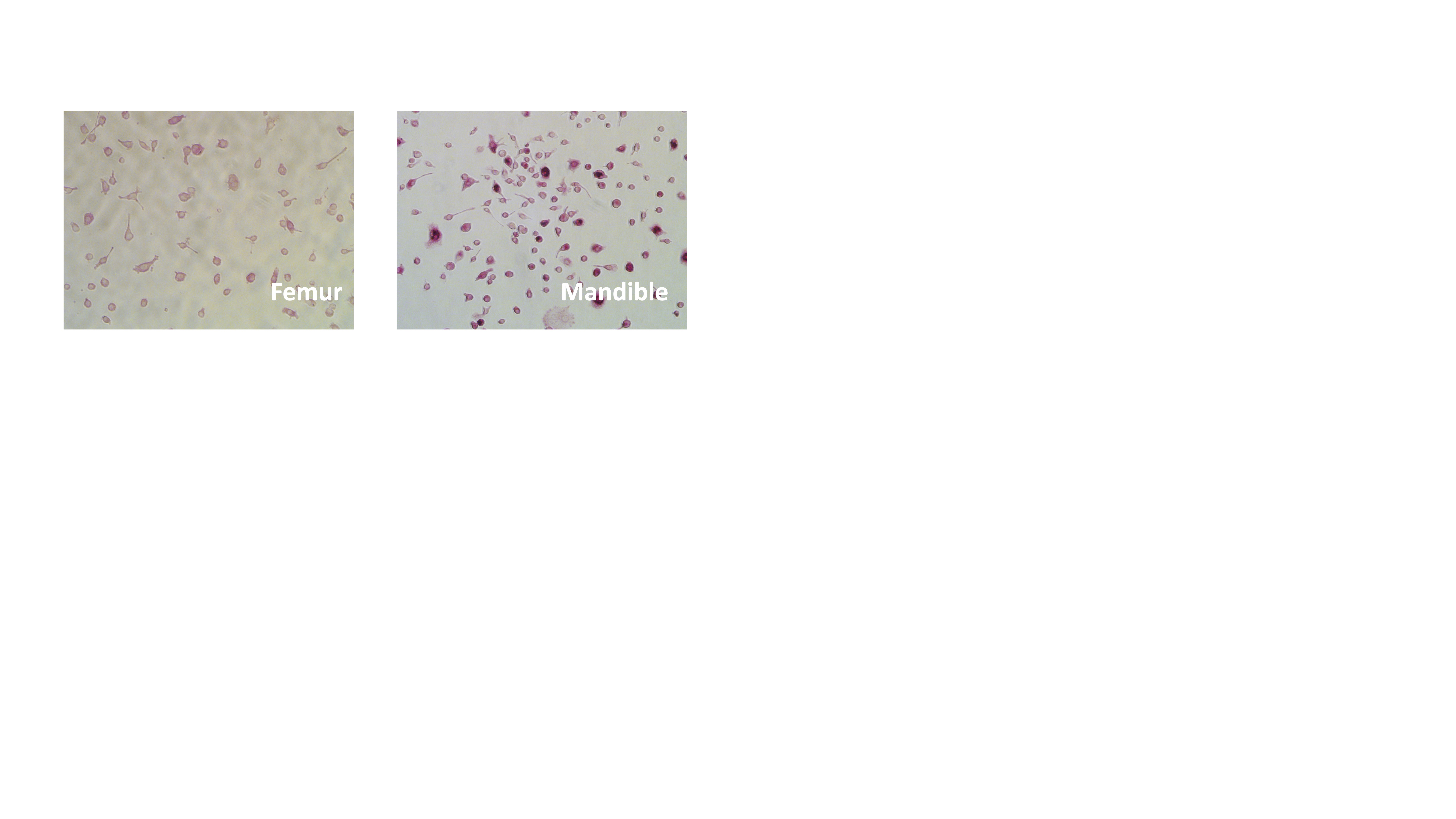

Supplement: Supplemental_Figure_1_for_JBMR_Plus_ziae029 [file supplemental_figure_1_for_jbmr_plus_ziae029.jpeg]

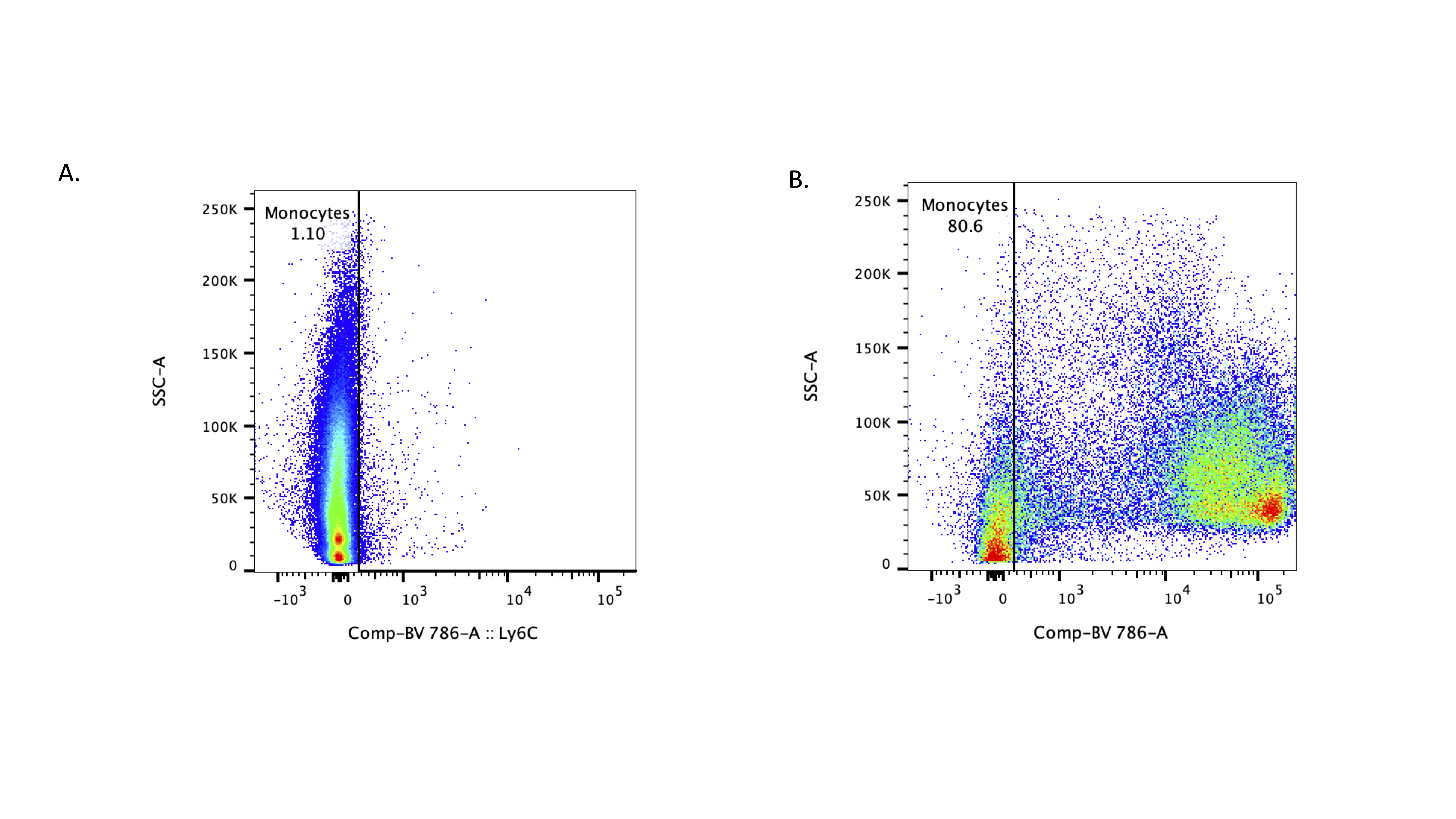

Supplement: Supplemental_figure_2_for_JBMR_Plus_ziae029 [file supplemental_figure_2_for_jbmr_plus_ziae029.jpeg]

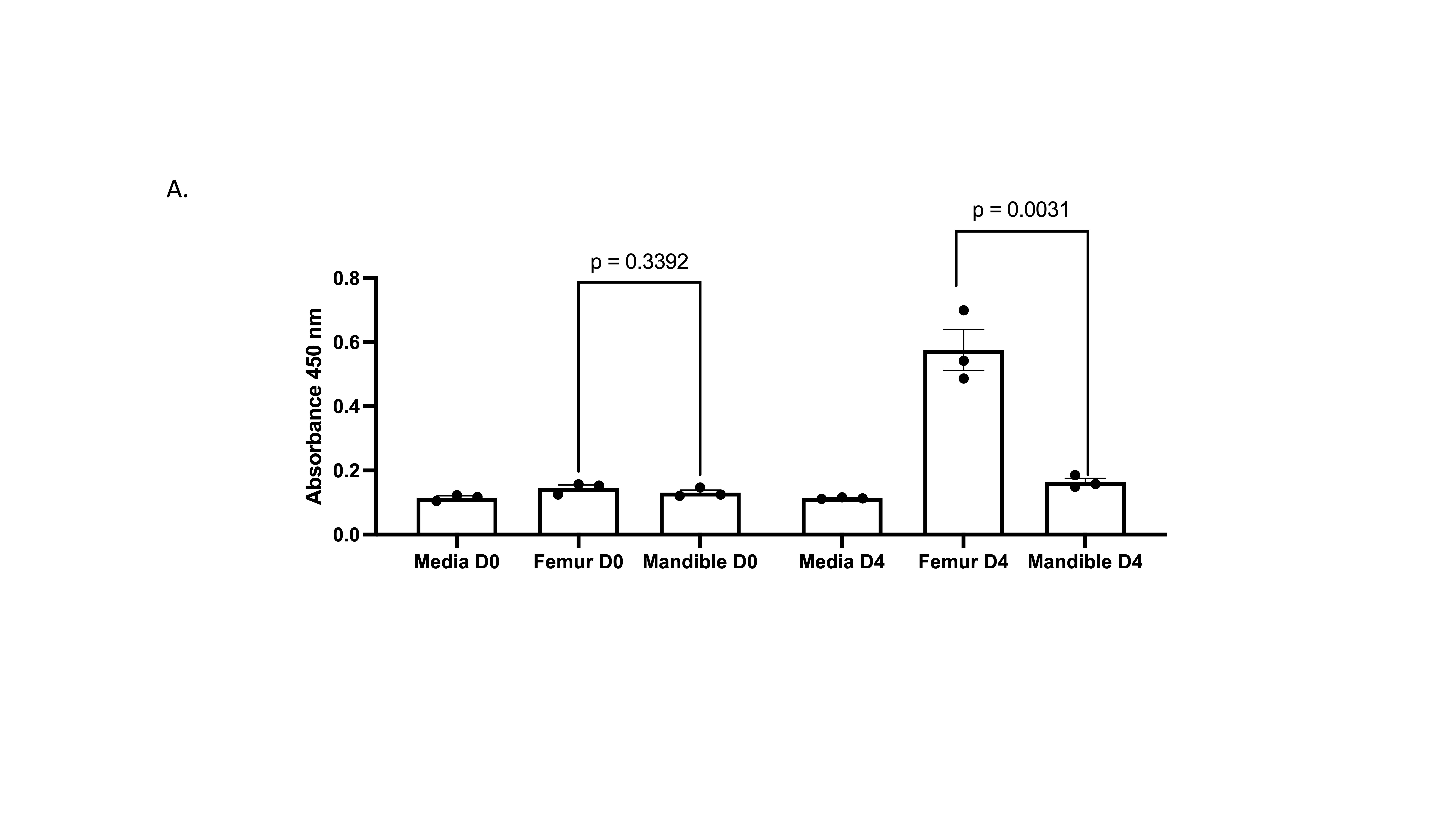

Supplement: Supplemental_figure_3_for_JBMR_Plus_ziae029 [file supplemental_figure_3_for_jbmr_plus_ziae029.jpeg]
